# Baicalin mitigates hyperglycemia-linked intestinal epithelial barrier impairment in part by inhibiting the formation of neutrophil extracellular traps

**DOI:** 10.3389/fimmu.2025.1551256

**Published:** 2025-03-03

**Authors:** Yiqing Cai, Qinbo Yang, Xinmiao Tang, Peiwei Wang, Jingang Cui, Xiaoye Du, Teng Zhang, Yu Chen

**Affiliations:** ^1^ Yueyang Hospital of Integrated Traditional Chinese and Western Medicine, Shanghai University of Traditional Chinese Medicine, Shanghai, China; ^2^ Clinical Research Institute of Integrative Medicine, Shanghai Academy of Traditional Chinese Medicine, Shanghai, China; ^3^ Laboratory of Clinical and Molecular Pharmacology, Yueyang Hospital of Integrated Traditional Chinese and Western Medicine, Shanghai University of Traditional Chinese Medicine, Shanghai, China

**Keywords:** hyperglycemia, intestinal epithelial barrier, neutrophils, neutrophil extracellular traps, baicalin

## Abstract

**Background:**

Under hyperglycemic conditions, impaired intestinal barrier integrity leads to heightened level of inflammation, playing important roles in driving diabetic complications. Emerging evidence supports the implications of neutrophil extracellular traps (NETs) in the pathogenesis of diabetes. However, whether NETs contribute to hyperglycemia-linked intestinal barrier impairment remains to be investigated. Moreover, baicalin, the major chemical component of Scutellaria baicalensis Georgi, is equipped with twofold intestinal protective and neutrophil suppressive activities. Yet, it is unclear if baicalin is effective at mitigating hyperglycemia-linked NETs-mediated intestinal barrier impairment.

**Methods:**

To directly address the mechanistic implications of NETs in hyperglycemia-linked intestinal epithelial barrier impairment, the impact of DNase I treatment or *Padi4* gene deficiency on intestinal epithelial integrity was first examined in the streptozotocin (STZ)-induced hyperglycemic mice *in vivo*. Next, the pharmacological impact of baicalin on NETs formation and intestinal epithelial barrier impairment was investigated in high glucose- and/or lipopolysaccharides (LPS)-stimulated neutrophils *in vitro* and in STZ-induced hyperglycemic mice *in vivo*, respectively.

**Results:**

The *in vitro* experiments confirmed that high glucose and/or LPS induced NETs formation. NETs directly impaired the viability and tight junction of the intestinal epithelial cells. The histological and immunohistochemical examinations unveiled that along with impaired intestinal epithelial morphology, citrullinated histone H3 (H3Cit), a marker of NETs, and neutrophil specific Ly6G were readily detected in the intestinal epithelium in the hyperglycemic mice. Without affecting the presence of neutrophils, DNase I treatment or *Padi4* gene deficiency markedly mitigated intestinal NETs formation and improved the intestinal morphology in the hyperglycemic mice. Notably, baicalin suppressed NETs formation and inhibited histone H3 citrullination stimulated by high glucose, LPS or both *in vitro*. Furthermore, baicalin blunted NETs formation and partially preserved the integrity of the intestinal epithelium in the hyperglycemic mice *in vivo*.

**Conclusions:**

The current study sheds new light on the pathophysiological implications of NETs in intestinal epithelial barrier impairment under hyperglycemic conditions. Most importantly, the findings here demonstrate for the first time that baicalin directly inhibits NETs formation stimulated by high glucose and/or LPS, which may in part account for its pharmacological effects at protecting against hyperglycemia-linked intestinal epithelial barrier impairment.

## 1 Introduction

Chronic hyperglycemia leads to the development of complications in multiple organs, accounting for diabetes-associated morbidities and mortalities. Systemic inflammation runs through the course of diabetes and promotes the development of diabetic complications (1). Hyperglycemia-associated systemic inflammation is in part caused by intestinal barrier impairment, which directly leads to aberrant translocation of the intestinal microbial contents into the bloodstream, triggering innate immune responses that augment the level of inflammation (2, 3). Understanding the mechanisms underlying hyperglycemia-linked intestinal barrier impairment may help to develop treatments to lower systemic inflammation under hyperglycemic conditions.

Neutrophils, the first-line defense of the innate immune system, contribute substantially to inflammation and host defense. Once activated, neutrophils execute the function of entrapping and killing exogenous pathogens by releasing neutrophil extracellular traps (NETs) consisting of nucleic acids, histones and granular proteins (4, 5). In addition to the canonical roles in host defense, the pathophysiological significance of NETs in the pathogenesis of systemic disorders such as diabetes has been gaining increased attention. Elevated circulating levels of NETs have been reported in the diabetic patients, correlating positively with inflammation as well as renal and cardiovascular complications in the patients. Moreover, high glucose directly induces NETs formation and the neutrophils from the diabetic patients and hyperglycemic mice are primed to produce NETs. Available evidence has also demonstrated that NETs play an indispensable role in promoting wound healing impairment and retinopathy under hyperglycemic conditions (6–10). It is also worth noting that altered intestinal homeostasis initiates transmigration of circulating neutrophils into the intestinal mucosa, leading to increased neutrophil infiltration and NETs formation in the intestinal epithelium. NETs can cause direct damage to the intestinal epithelial cells, impairing the integrity of the intestinal epithelial barrier (11). However, whether NETs formation is implicated in hyperglycemia-linked intestinal epithelial barrier impairment remains unknown.

Baicalin is a glycosidic flavone abundantly present in Scutellaria baicalensis Georgi, a herb that has long been used for the treatment of inflammation-related diseases and intestinal disorders in traditional Chinese medicine (12–15). Baicalin alleviates the intestinal barrier impairment under conditions such as hypertension and inflammation (16, 17). Moreover, baicalin suppresses neutrophil activation induced by phorbol-12-myristate-13-acetate (PMA) or N-formyl-methionyl-leucyl-phenylalanine (fMLP) (18). However, the pharmacological implications of baicalin in protecting against hyperglycemia-associated intestinal epithelial barrier impairment are unclear. Moreover, the impact of baicalin on high glucose-induced NETs formation and potential hyperglycemia-associated intestinal NETs formation remains to be addressed.

Therefore, based on addressing the implications of NETs in hyperglycemia-linked intestinal epithelial barrier impairment, the current study investigated the pharmacological potential and mechanisms of baicalin in attenuating hyperglycemia-associated NETs formation and intestinal epithelial barrier impairment.

## 2 Materials and methods

### 2.1 Neutrophil isolation and NETs formation assay

Neutrophils were isolated using a Neutrophil Isolation Kit (P8550, Solarbio, China) and maintained at 37°C in a humidified incubator with 5% CO_2_. Briefly, after removing the erythrocytes from the bone marrow cells collected from the femurs and tibias, the neutrophil isolation reagent was applied, followed by centrifugation at 900 g for 30 min. The isolated neutrophils were seeded at 2×10^5^ cells per well in 48-well plates in low-glucose (1 g/L glucose) or high-glucose (4.5 g/L glucose) DMEM medium (11965, Gibco, USA) for 2.5 h in the presence or absence of 10 μg/mL LPS (L2630, Sigma-Aldrich, USA). For the experiments involving baicalin treatment, baicalin was added to the high-glucose-stimulated neutrophils at 25, 50 or 100 μM or the neutrophils stimulated by LPS in both low-glucose and high-glucose culture conditions at 100 μM, followed by incubation at 37°C for 2.5 h in a humidified incubator with 5% CO_2_. To assess NETs formation, the neutrophils were fixed in 4% paraformaldehyde at room temperature for 10 min, permeabilized using 0.1% Triton X-100 in PBS at room temperature for 10 min and blocked with 10% donkey serum at room temperature for 1 h. The cells were then stained with rabbit anti-histone H3 (citrulline R2 + R8 + R17) antibody (1:500, ab5103, Abcam, USA) at room temperature for 2 h and Alexa Fluor 488 donkey anti-rabbit IgG-H&L secondary antibody (1:1000, ab150073, Abcam, USA) in the dark at room temperature for 1 h, respectively. Nuclei were counterstained using 4-6-diamidino-2-phenylindole (DAPI) (10236276001, Roche, Germany). Images were acquired using a fluorescence microscope (DMI6000, Leica, Germany) and the imaging settings were kept the same for all the images. Citrullinated histone 3 (H3Cit) positive area was quantified using Image J.

### 2.2 NETs isolation and quantification

The neutrophils were isolated as described above. Isolated neutrophils were seeded in 10-cm cell culture dishes at the number of 2×10^7^ per dish and cultured at 37°C in a humidified incubator with 5% CO_2_. NETosis was then induced by incubation with 500 nM PMA (P8139, Sigma-Aldrich, USA) for 4 h. Afterward, the culture medium was gently aspirated, leaving the layer of NETs and neutrophils adhered to the cell culture dishes. NETs and neutrophils were then dislodged and collected in cold PBS, followed by centrifugation at 450 g at 4°C for 10 min. The supernatants containing NETs were collected and the amount of DNA in the supernatants was measured using a Nanodrop (ThermoFisher Scientific, USA) or Quant-iT PicoGreen dsDNA Assay Kit (P11496, ThermoFisher Scientific, USA). The isolated NETs were stored at -80°C prior to the indicated experiments.

### 2.3 Cell culture and treatments

The rat small intestinal epithelial cell line IEC-6 (CRL-1592, ATCC, USA) was maintained in DMEM supplemented with 10% fetal bovine serum (10099-141, Gibco, USA), 100 unit/ml streptomycin (15140122, Gibco, USA) and 0.1 unit/mL bovine insulin at 37°C in a humidified incubator with 5% CO_2_. To assess the tight junction, IEC-6 cells were seeded in 48-well plates at the density of 1×10^5^ cells/well, followed by treatment of NETs at 25, 50, 100, 200 and 400 ng/mL for 12 h. The cells were then fixed in 4% paraformaldehyde at room temperature for 10 min, permeabilized with 0.1% Triton X-100 in PBS at room temperature for 10 min and blocked with 10% sheep serum at room temperature for 1 h. Afterwards, the cells were sequentially stained with the rabbit anti-ZO1 tight junction protein monoclonal antibody (1:500, ab221547, Abcam, USA) at room temperature for 2 h and sheep anti-rabbit IgG antibody, Cy3 conjugate (1:1000, AP510C, Sigma-Aldrich) at room temperature for 1 h. Nuclei were counterstained by DAPI. Images were acquired using a fluorescent microscope with the same imaging settings (DMI6000, Leica, Germany). Five or six non-overlapping images captured from each well were subjected to the analysis of the fluorescence intensity of ZO-1 using the Analyze Particles function in ImageJ. Moreover, tight junction organization rate (TiJOR) was assessed following the previously described method (19). For cell viability assessment using thiazolyl blue tetrazolium bromide (MTT) (M8180, Solarbio, China), IEC-6 cells were seeded in 96-well plates at the density of 1×10^4^ cells/well and treated by NETs at 25, 50, 100, 200 and 400 ng/mL for 12 h. After the indicated treatments, the cells were incubated in the presence of MTT reagent (50 μg/well) at 37°C for 4 h, followed by the measurement of the absorbance at 490 nm using a plate reader (Epoch, BioTek, USA).

### 2.4 Western blotting

The neutrophils were snap frozen and homogenized in RIPA buffer (P0013C, Beyotime, China) supplemented with protease inhibitor cocktails (04693116001, Roche, Germany). Electrophoresis was performed in 15% SDS-PAGE gels at room temperature, which was started at a constant voltage of 80 V. When the bromophenol blue indicator formed a straight line and the molecular weight markers were separated, the voltage was adjusted to 100 V and the electrophoresis was carried out at a constant voltage of 100 V for additional 2 h. Afterwards, the protein was transferred to polyvinylidene fluoride membrane (IPVH85R, Millipore, USA) in an ice pack for 90 min under a constant current of 200 mA. The levels of H3Cit and histone H3 were examined using rabbit anti-histone H3 (citrulline R2 + R8 + R17) antibody (1:1000, ab5103, Abcam, USA) and rabbit anti-histone H3 polyclonal antibody (1:1000, ab1791, Abcam, USA) at room temperature for 2 h, respectively. Goat anti-rabbit IgG (H+L)-HRP conjugate secondary antibody (1:3000, W4011, Promega, USA) was applied at room temperature for 1 h following the primary antibody incubation. The blots were developed with enhanced chemiluminescence substrate (34096, ThermoFisher Scientific, USA) and the images were acquired using an UVP imaging system (BioSpectrum 410, USA). The densitometry of the protein bands was performed using ImageJ.

### 2.5 Animals and treatments

Male C57BL/6 mice at 6-8 weeks of age were purchased from Shanghai Laboratory Animal Co., Ltd., China. *Padi4*-/- mice in the C57BL/6 background (NM-KO-190334) were ordered from Shanghai Model Organisms, China. The animals were maintained under controlled temperature (20 ± 2°C), humidity (35-55%) and lighting (12 h light/dark cycle) conditions with free access to food and water in the animal resources facility at Yueyang Hospital of Integrated Traditional Chinese and Western Medicine, Shanghai University of Traditional Chinese Medicine. Two-week acclimatization was allowed prior to further treatments. All animal procedures were approved by the Institutional Animal Care and Use Committee of Yueyang Hospital of Integrated Traditional Chinese and Western Medicine, Shanghai University of Traditional Chinese Medicine (YYLAC-2019-044). All animal experiments were in compliance with the ARRIVE guidelines and conducted following National Research Council’s Guide for the Care and Use of Laboratory Animals. To induce hyperglycemia, streptozotocin (STZ) (S8050, Solarbio, China), freshly prepared in sodium citrate buffer (pH 4.4), was intraperitoneally injected at 50 mg/kg/day for 5 consecutive days. Normal controls received the vehicle injection in the same manner. One week after the last injection of STZ, fasting blood glucose was measured using a blood glucose meter (Contour TS, Bayer, Germany). Hyperglycemia was defined by significantly elevated fasting blood glucose compared to the normal controls without STZ administration. Baicalin (B20570, Yuanye Biotechnology Co., Ltd, Shanghai, China) was prepared in 0.5% sodium carboxymethyl cellulose solution (vehicle). Hyperglycemic mice received the 2-week daily treatment of either vehicle or baicalin at 240 mg/kg or 1200 mg/kg via oral gavage. Normal controls were given the vehicle solution in the same manner. Or DNase I (D8071, Solarbio, China), dissolved in saline, was administered to the hyperglycemic mice at the daily dose of 2.5 mg/kg or 10 mg/kg via oral gavage for 2 weeks. Body weight and fasting blood glucose were measured before and after the indicated treatments.

### 2.6 Histological examination

The ileal specimens were fixed in 4% paraformaldehyde at room temperature for 24 h and processed for paraffin embedding and sectioning. Ileal sections (4 μm thick) were subjected to hematoxylin & eosin (HE) or Alcian Blue staining. Micrographs were acquired using a light microscope (DM2000, Leica, Germany). The number of goblet cells was quantified using ImageJ.

### 2.7 Immunohistochemistry

The ileal paraffin sections were deparaffinized and antigen-retrieved in 10 mM sodium citrate (pH 6.0) prior to the detection of cingulin, H3Cit and Ly6G using rabbit anti-cingulin polyclonal antibody (1:500, NBP1-89600, Novus, USA), rabbit anti-histone H3 (citrulline R2 + R8 + R17) antibody (1:500, ab5103, Abcam, USA) and rat anti-mouse Ly6G monoclonal antibody (1:250, 551461, BD Pharmingen, USA) at room temperature for 2 h, respectively. The secondary antibodies included Alexa Fluor 488 donkey anti-rabbit IgG-H&L (1:500, ab150073, Abcam, USA), Cy3 sheep anti-rabbit IgG (whole molecule)-F(ab′)2 fragment (1:1000, Sigma-Aldrich, C2306, MO, USA) and Cy3 goat anti-rat IgG-H&L (1:500, ab98416, Abcam, USA). The secondary antibody incubation was performed at room temperature for 2 h. Counterstaining with DAPI was performed to visualize the nuclei. The fluorescent images were acquired using a fluorescence microscope with the imaging parameters (e.g., exposure and gain) kept the same for each marker (DM6000B, Leica, Germany).

### 2.8 Statistics analysis

Data are expressed as mean ± SEM. Statistical analyses were performed using the Graphpad Prism 9 software. A two-tailed Student’s t-test was performed for comparisons between two groups. One-way ANOVA was performed to compare the means of three or more experimental groups. *Post-hoc* test with Tukey correction or Mann-Whitney U test was applied to multiple comparisons. Statistical significance was defined as p < 0.05.

## 3 Results

### 3.1 NETs impair the tight junction and viability of the intestinal epithelial cells

**Figure 1:**
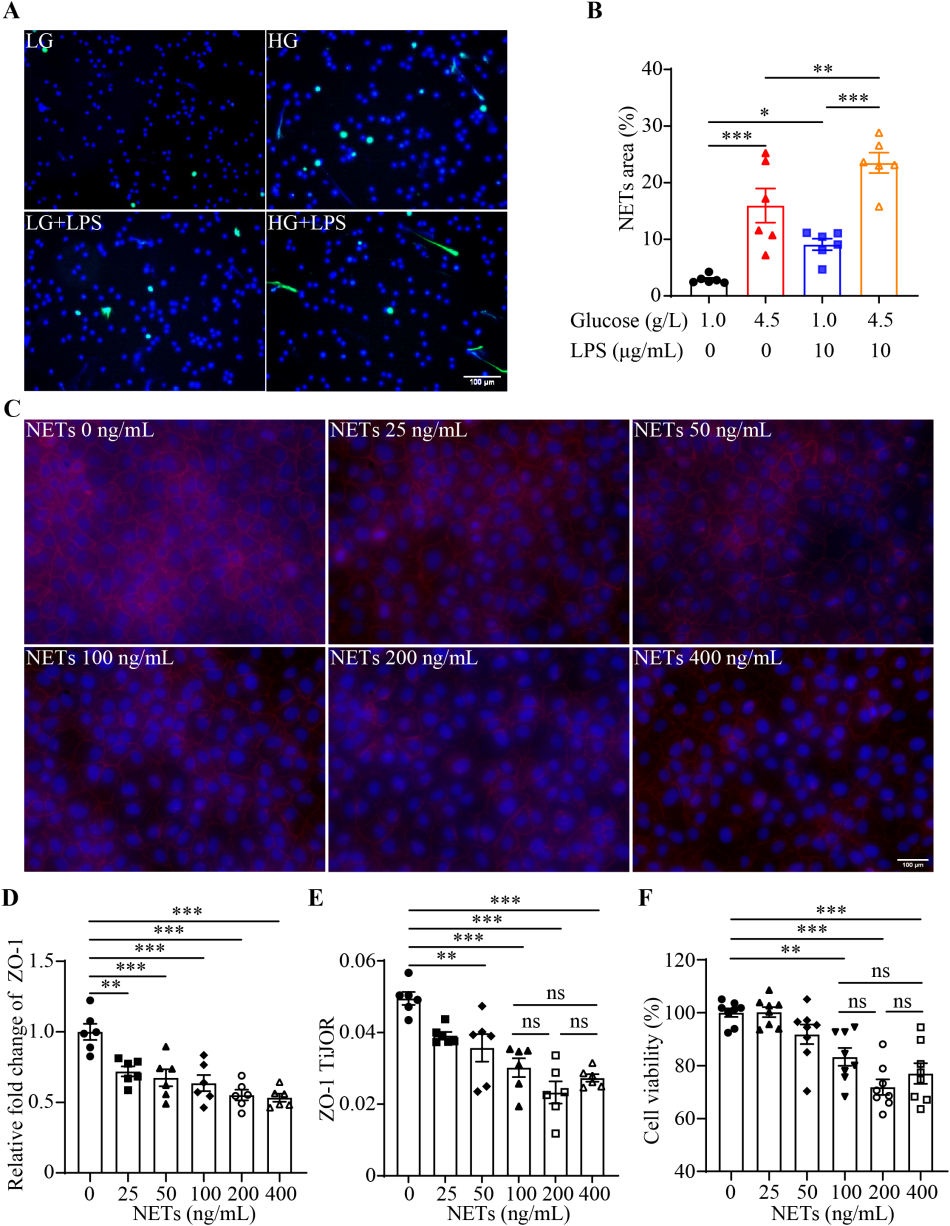
High glucose and/or LPS induces NETs formation and NETs impair the tight junction and viability of the intestinal epithelial cells. **(A)** Isolated mouse neutrophils were exposed to 1 g/L glucose (LG), 4.5 g/L glucose (HG), 1 g/L glucose along with 10 ug/mL (LG+LPS) or 4.5 g/L glucose along with 10 ug/mL LPS (HG+LPS) for 2.5 h, followed by the assessment of the H3Cit positivity (in green). Nuclei were visualized by DAPI counterstaining (in blue). Scale bar, 100 μm. **(B)** NETs were quantified as the percentage of H3Cit positive area (n=6 per group). **(C)** IEC-6 cells were stimulated with NETs at 25, 50, 100, 200 or 400 ng/mL for 12 h, followed by the assessment of the ZO-1 positivity (in red). Nuclei were visualized by DAPI counterstaining (in blue). Scale bar, 100 μm. **(D)** Quantification of the ZO-1 positivity (n = 6 per group). **(E)** Quantification of the TiJOR for ZO-1 (n = 6 per group). **(F)** The viability of IEC-6 cells was examined by MTT assay 12 h after NETs stimulation at 25, 50, 100, 200 or 400 ng/mL (n = 8 per group). Data were expressed as mean ± SEM. * p < 0.05, ** p < 0.01, *** p < 0.001, ns, not significant.

Prior to validating the impact of NETs on the barrier integrity and survival of the intestinal epithelial cells, the effects of high glucose on the formation of NETs in the presence or absence of LPS were confirmed. H3Cit was adopted to mark the formation of NETs after the indicated treatments. Measurement of the area of NETs revealed increased formation of NETs in the neutrophils exposed to either high glucose or LPS. Meanwhile, a further increase in the NETs formation was found in the neutrophils exposed to both high glucose and LPS (**Figures 1A, B**). Next, IEC-6 intestinal epithelial cells were incubated with vehicle or NETs at 25, 50, 100, 200 or 400 ng/mL. Diminished ZO-1 immunopositivity was observed in the NETs-exposed IEC-6 cells at all the doses examined (**Figures 1C, D**). Additionally, significantly decreased TiJOR was associated with the cells incubated with 50, 100, 200 or 400 ng/mL NETs, suggesting that the tight junction network was disorganized as a result of NETs exposure (**Figure 1E**). Meanwhile, the survival of IEC-6 was remarkedly compromised in the presence of relatively higher doses of NETs (e.g., 100, 200 or 400 ng/mL). The deleterious impact of NETs on the survival of the IEC-6 cells appeared to reach a plateau at around 100 ng/mL since no statistical significance was noted among the cells treated with 100 ng/mL NETs, 200 ng/mL NETs and 400 ng/mL NETs (**Figure 1F**). These results confirm that high glucose promotes NETs production and NETs directly impair the barrier integrity of the intestinal epithelial cells, suggesting potential contribution of NETs formation in hyperglycemia-linked intestinal epithelial barrier impairment.

### 3.2 Hyperglycemia leads to morphological impairment and NETs formation in the intestinal epithelium

**Figure 2:**
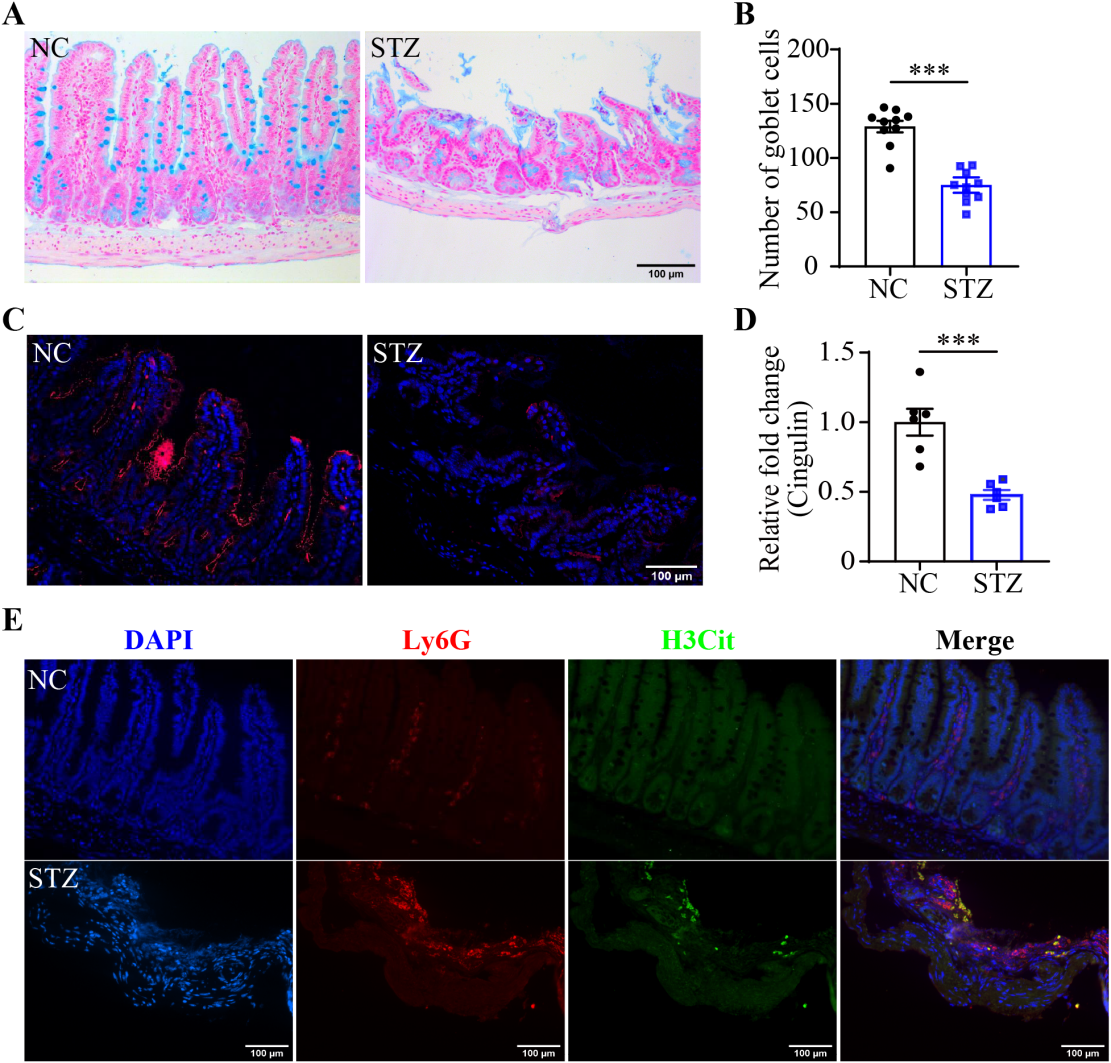
Hyperglycemia leads to mucosal barrier impairment and NETs formation in the intestinal epithelium. **(A)** Representative micrographs of Alcian blue-stained goblet cells in the ileum. Scale bar, 100 μm. **(B)** The number of goblet cells per unit area (n = 10 per group). **(C)** Representative micrographs of cingulin immunopositivity (in red). The nuclei were counterstained by DAPI (in blue). Scale bar, 100 μm. **(D)** Cingulin immunopositivity per unit area (n = 6 per group). **(E)** Representative images of Ly6G (in red) and H3Cit (in green) immunopositivity. DAPI was counterstained to visualize the nuclei (in blue). Scale bar, 100 μm. Data were expressed as mean ± SEM. *** p < 0.001. NC, normoglycemic controls; STZ, STZ-induced hyperglycemic mice.

Next, the possible implication of NETs formation in hyperglycemia-linked intestinal barrier impairment was explored. HE staining showed that compared to the intact ileal morphology detected in the normal controls, leukocyte infiltration, capillary dilation and congestion in the villi, villus shedding and necrosis, and focal mucosal ulceration were readily detected in the intestinal mucosa and submucosa from the STZ mice (**Supplementary Figure 1**). Alcian blue staining revealed that the number of the goblet cells, the essential component of the intestinal epithelial barrier, was significantly reduced in the STZ mice compared to the normal controls (**Figures 2A, B**). IHC demonstrated that the immunopositivity cingulin, which regulates tight junction permeability, was orderly distributed around the villus and crypts in the ilium from the normal control. In contrast, remarkably diminished cingulin immunopositivity was observed in the ileum from the STZ mice (**Figures 2C, D**). Furthermore, overt immunopositivity of Ly6G, a neutrophil marker, was readily detected in the ilium from the STZ mice but not the normal controls. Moreover, H3Cit immunopositivity was found to be colocalized with Ly6G positive neutrophils in the ilium from the STZ mice (**Figure 2E**). These results indicate that hyperglycemia leads to barrier integrity impairment and NETs formation in the intestinal epithelium.

### 3.3 NETs promote hyperglycemia-associated intestinal epithelial barrier impairment

**Figure 3:**
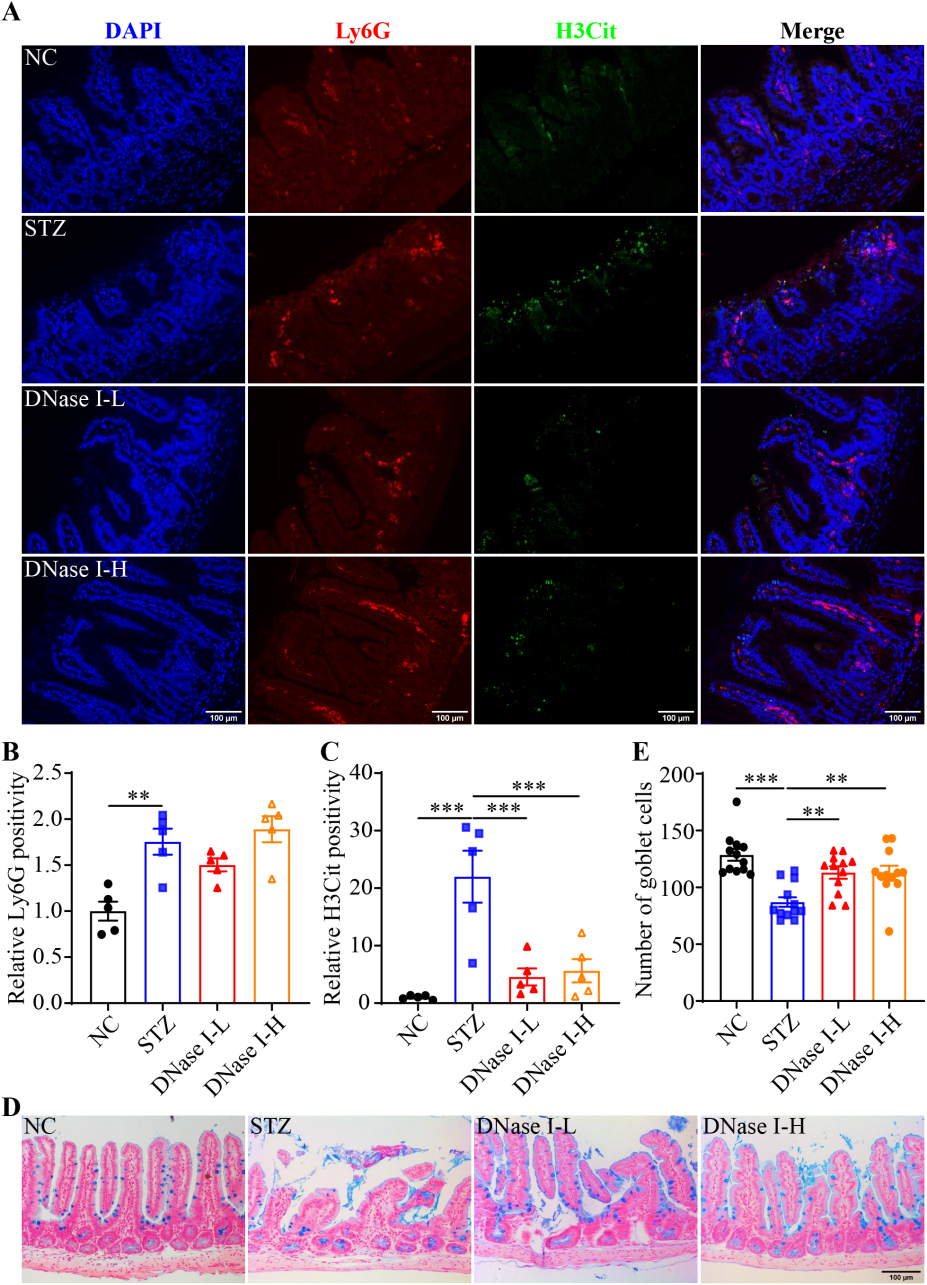
DNase I treatment attenuates intestinal NETs formation and mucosal barrier impairment in the hyperglycemic mice. **(A)** Representative micrographs showing Ly6G immunopositivity (in red), H3Cit immunopositivity (in green) and DAPI positivity (in blue) in the ileal sections. Scale bar, 100 μm. **(B)** Relative Ly6G positivity (n = 5 per group). **(C)** Relative H3Cit positivity (n = 5 per group). **(D)** Representative images of Alcian blue-stained goblet cells. Scale bar, 100 μm. **(E)** The number of goblet cells per unit area (n = 12 per group). Data were expressed as mean ± SEM. ** p < 0.01, *** p < 0.001. NC, the vehicle-treated normoglycemic controls; STZ, the STZ-induced hyperglycemic mice treated with vehicle; DNase I-L, the STZ-induced hyperglycemic mice treated with a daily dose of 2.5 mg/kg DNase I; DNase I-H, the STZ-induced hyperglycemic mice treated with a daily dose of 10 mg/kg DNase I.

**Figure 4:**
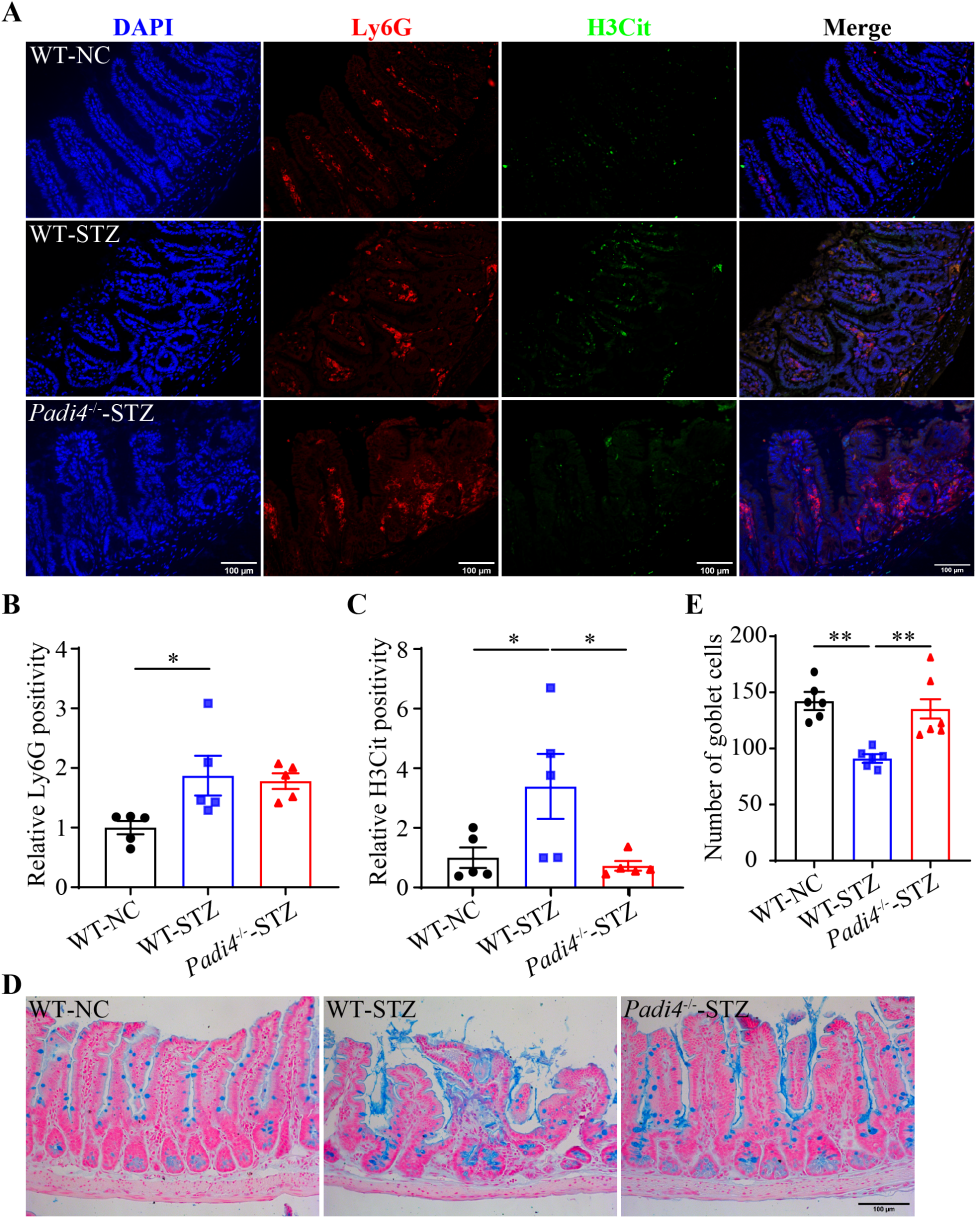
*Padi4* gene deficiency mitigates intestinal NETs formation and mucosal barrier impairment in the hyperglycemic mice. **(A)** Representative micrographs showing Ly6G immunopositivity (in red), H3Cit immunopositivity (in green) and DAPI positive nuclei (in blue) in the ileal sections. Scale bar, 100 μm. **(B)** Relative Ly6G positivity (n = 5 per group). **(C)** Relative H3Cit positivity (n = 5 per group). **(D)** Representative images of Alcian blue-stained goblet cells. Scale bar, 100 μm. **(E)** The number of goblet cells per unit area (n = 6 per group). Data were expressed as mean ± SEM. * p < 0.05, ** p < 0.01. WT-NC, the wild type normoglycemic controls; WT-STZ, the wild type mice subjected to STZ-induced hyperglycemia; *Padi4*-/–STZ, the *Padi4*-/- mice subjected to STZ-induced hyperglycemia.

To further clarify the causal relationship between NETs and hyperglycemia-linked intestinal barrier impairment, DNase I was administered to the hyperglycemic mice to degrade the DNA backbone of NETs. Compared to the vehicle-treated STZ mice, no significant changes in the fasting blood glucose were observed in the DNase I-treated STZ mice (**Supplementary Figure 2A**). IHC revealed significantly diminished H3Cit immunopositivity but not Ly6G immunopositivity in the ilium from the STZ mice treated with DNase I at both 2.5 and 10 mg/kg (**Figures 3A–C**). The ileal morphology was partially preserved in the DNase-treated STZ mice compared to the vehicle-treated STZ mice (**Supplementary Figure 2B**). Moreover, the number of goblet cells was significantly higher in the DNase-treated STZ mice compared to the vehicle-treated STZ mice (**Figures 3D, E**). These observations support a causal relationship between NETs and intestinal epithelial barrier impairment under hyperglycemic conditions. To further validate this causality, *Padi4*-/- mice, which are deficient in the gene encoding PAD4, an essential driver of NETs formation, were subjected to STZ-induced hyperglycemia. Decreased immunopositivity of H3Cit but not that of Ly6G was observed in the hyperglycemic *Padi4*-/- mice compared to the hyperglycemic wild type controls (**Figures 4A–C**). Meanwhile, similar to DNase I treatment, no significant changes in the fasting blood glucose were observed in the *Padi4*-/- mice. However, improved ileal morphology was observed in the hyperglycemic *Padi4*-/- mice compared to the hyperglycemic wild type controls (**Supplementary Figures 3A, B**). Moreover, compared to the hyperglycemic wide type controls, the number of goblet cells was significantly increased in the hyperglycemic *Padi4*-/- mice (**Figures 4D, E**). These results collectively indicate that it is the NETs formation but not the mere presence of neutrophils that causes the intestinal epithelial barrier impairment under hyperglycemic conditions.

### 3.4 Baicalin suppresses NETs formation induced by high glucose, LPS or both high glucose and LPS

**Figure 5:**
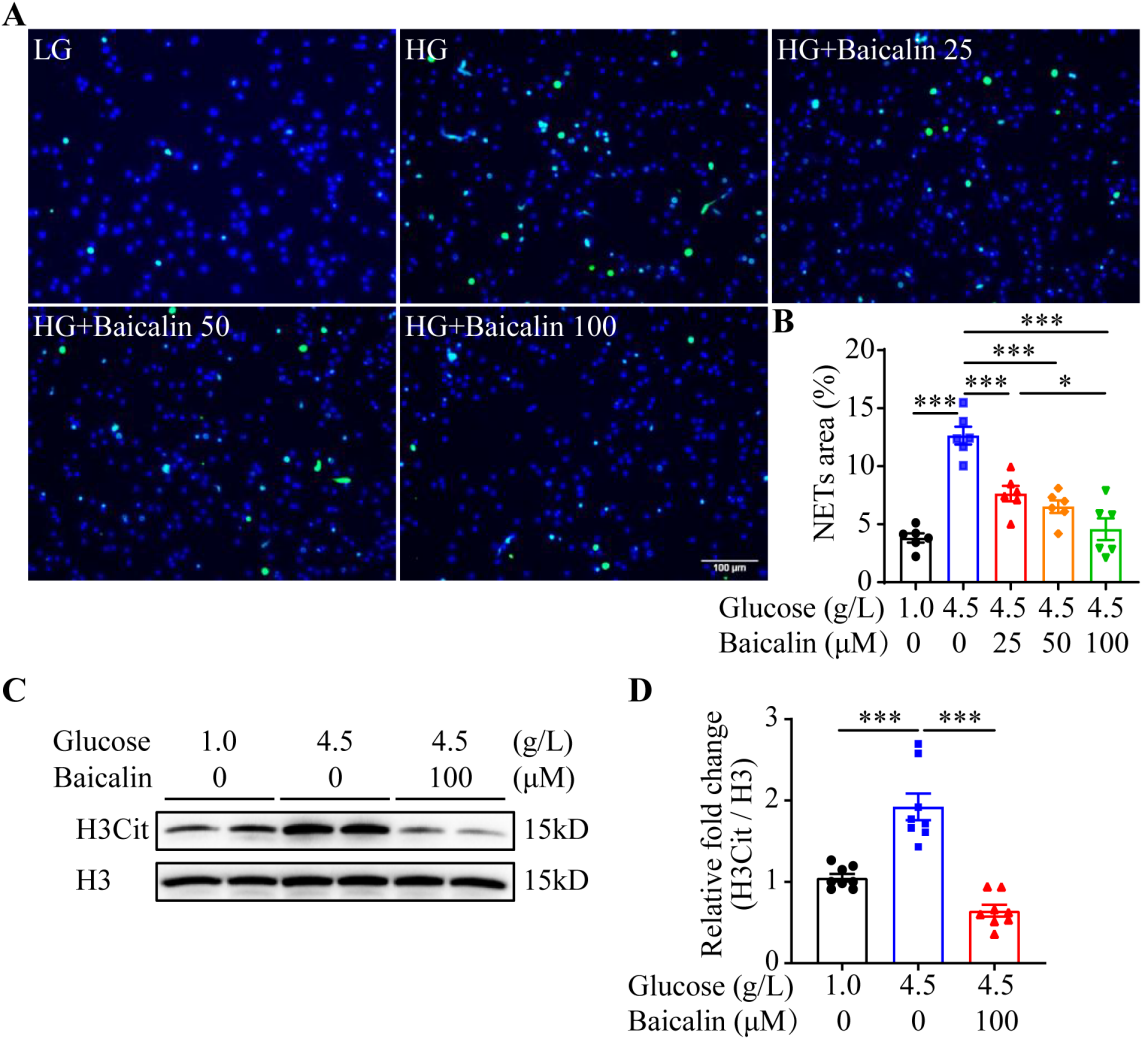
Baicalin suppresses high glucose-induced NETs formation. **(A)** The isolated mouse neutrophils were exposed to 1 g/L glucose (LG) or 4.5 g/L glucose (HG). The HG-exposed neutrophils were treated with either vehicle or baicalin at 25, 50, 100 μM for 2.5 h, followed by the assessment of H3Cit immunopositivity. Representative images showing H3Cit immunopositivity. Scale bar, 100 μm. **(B)** NETs formation was quantified as the percentage of the H3Cit positive area (n = 6 per group). **(C)** Isolated neutrophils were exposed to 1 g/L glucose or 4.5 g/L glucose. The cells exposed to 4.5 g/L glucose were treated with vehicle or 100 μM baicalin for 2.5 h, followed by the evaluation of the level of histone H3 citrullination. Histone H3 served as the loading control. **(D)** Relative fold change in the level of H3Cit (n = 8 per group). Data were expressed as mean ± SEM. * p < 0.05, *** p < 0.001.

**Figure 6:**
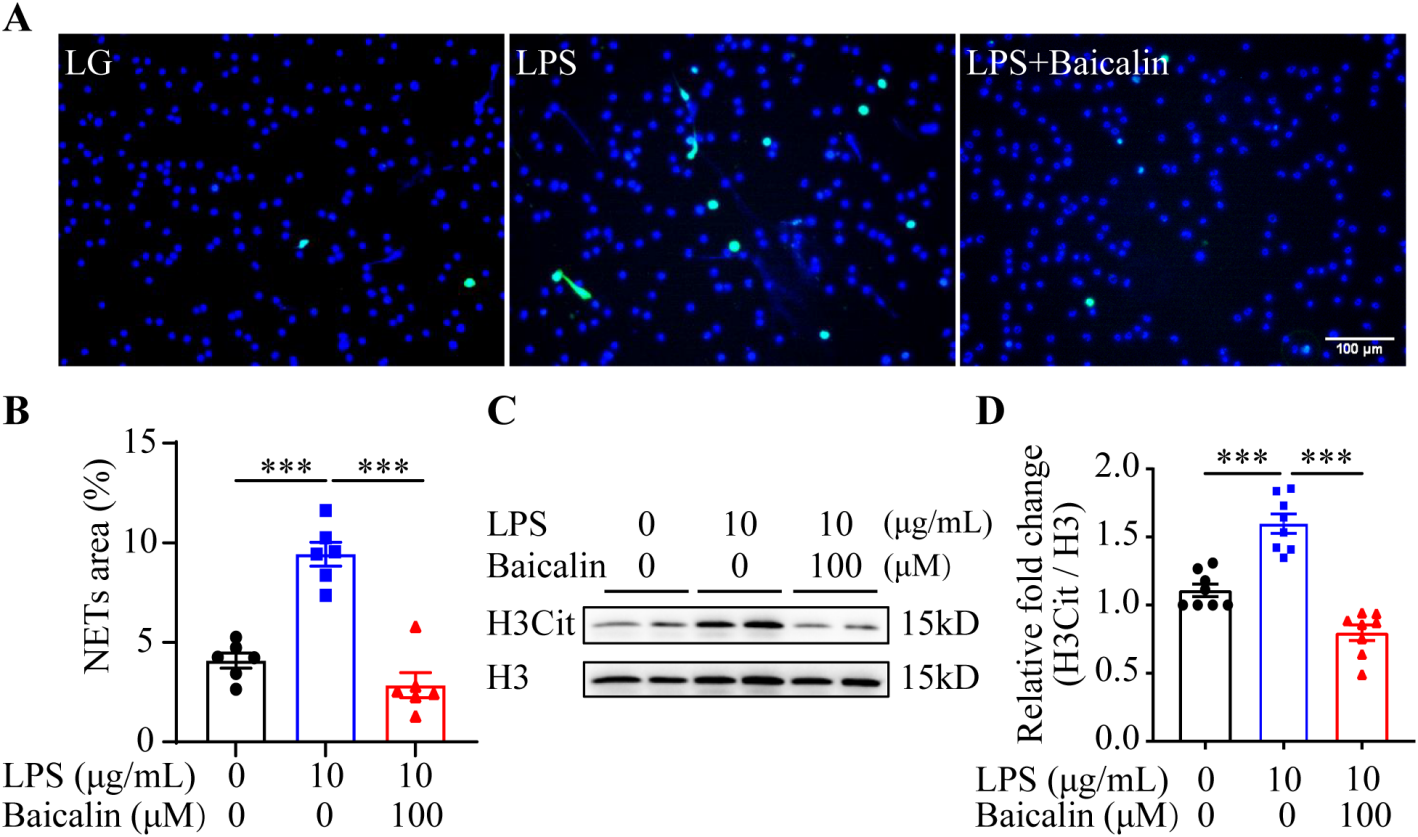
Baicalin inhibits LPS-induced NETs formation. **(A)** The isolated mouse neutrophils cultured in 1 g/L glucose were stimulated with 10 μg/mL LPS (LPS) in the presence or absence of 100 μM baicalin for 2.5 h, followed by the examination of the H3Cit immunopositivity. The cells unexposed to LPS and treated by vehicle (VC) served as the baseline for the indicated analysis. Representative immunofluorescent images showing H3Cit immunopositivity. Scale bar, 100 μm. **(B)** NETs formation was quantified as the percentage of the H3Cit positive area (n = 6 per group). **(C)** Histone H3 citrullination was assessed by examining the protein level of H3Cit. Histone H3 was probed as the internal control. **(D)** Relative fold change of H3Cit (n = 8 per group). Data were expressed as mean ± SEM. *** p < 0.001.

**Figure 7:**
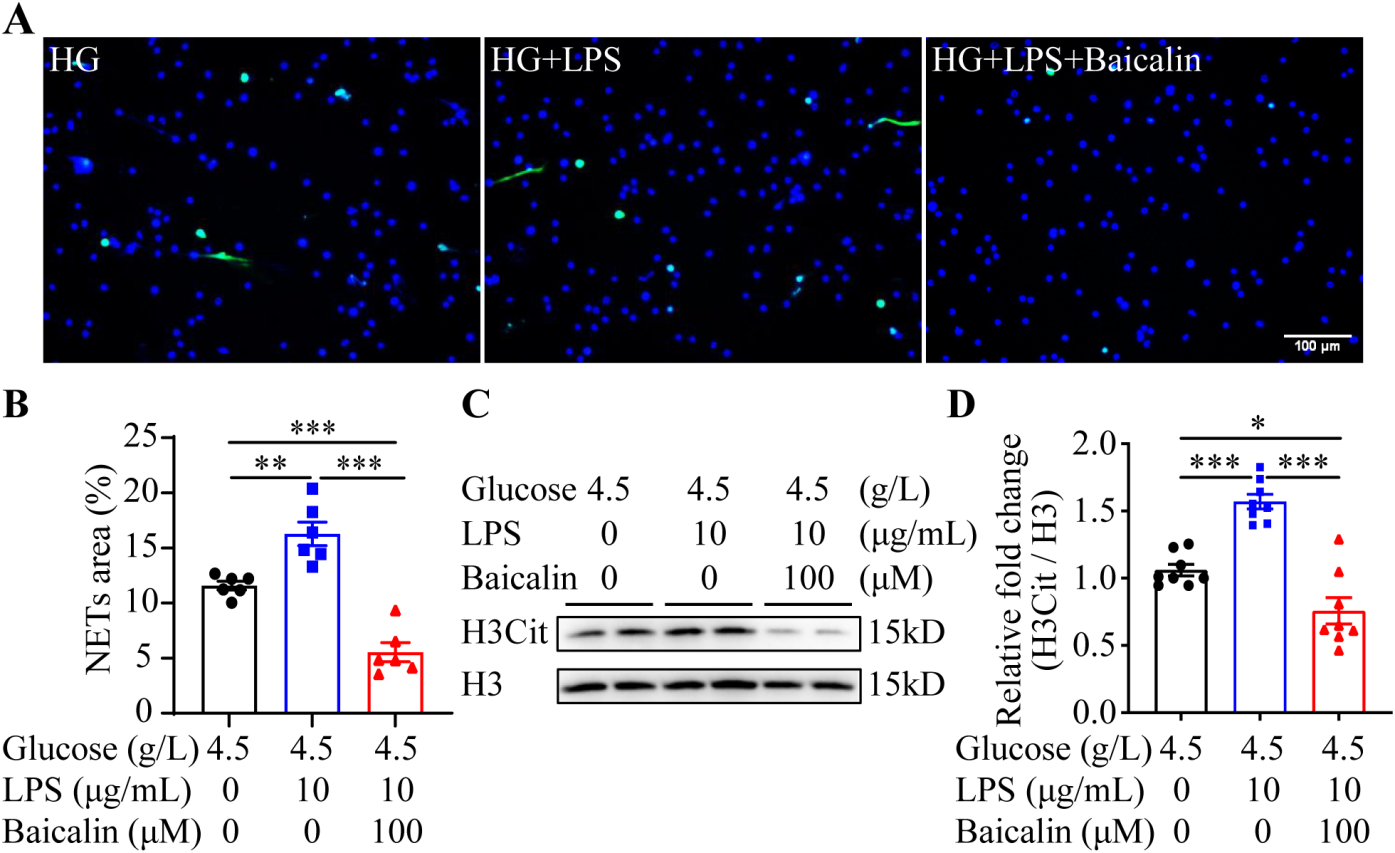
Baicalin inhibits NETs formation in the presence of both high glucose and LPS. **(A)** The isolated neutrophils were stimulated by 4.5 g/L glucose (HG) or both 4.5 g/L glucose and 10 μg/mL LPS (HG+LPS) in the presence or absence of 100 μM baicalin for 2.5 h, followed by the assessment of the H3Cit immunopositivity. Representative immunofluorescent images showing the H3Cit immunopositivity. Scale bar, 100 μm. **(B)** NETs formation was quantified as the percentage of the H3Cit positive area (n = 6 per group). **(C)** Histone H3 citrullination was assessed by examining the protein level of H3Cit. Histone H3 served as the internal control. **(D)** Relative fold change of H3Cit (n = 8 per group). Data were expressed as mean ± SEM. * p < 0.05, ** p < 0.01, *** p < 0.001.

The results above suggest the possibility that inhibiting NETs formation may mitigate hyperglycemia-linked intestinal epithelial barrier impairment. Available evidence supports the intestinal barrier protective activities of baicalin under hypertensive or inflammatory conditions (16, 17). Moreover, it has been shown that baicalin directly attenuates neutrophil activation (18). To further evaluate the pharmacological activity of baicalin in protecting against hyperglycemia-linked, NETs-mediated intestinal epithelial barrier impairment, we assessed the effects of baicalin on NETs formation in the neutrophils subjected to high glucose and/or LPS stimulation. The results showed that baicalin dose-dependently reduced high glucose-stimulated NETs formation (**Figures 5A, B**). Furthermore, high glucose-induced increases in H3Cit were significantly blunted in the baicalin-treated neutrophils (**Figures 5C, D**). In addition, baicalin attenuated NETs formation and histone citrullination in the neutrophils stimulated by LPS (**Figures 6A–D**) or in the neutrophils exposed to both high glucose and LPS (**Figures 7A–D**). These results demonstrate that baicalin is pharmacologically effective at suppressing histone citrullination, thereby attenuating NETs formation in the presence of high glucose, LPS or combination of high glucose and LPS.

### 3.5 Baicalin attenuates hyperglycemia-associated NETs formation and intestinal barrier impairment *in vivo*

**Figure 8:**
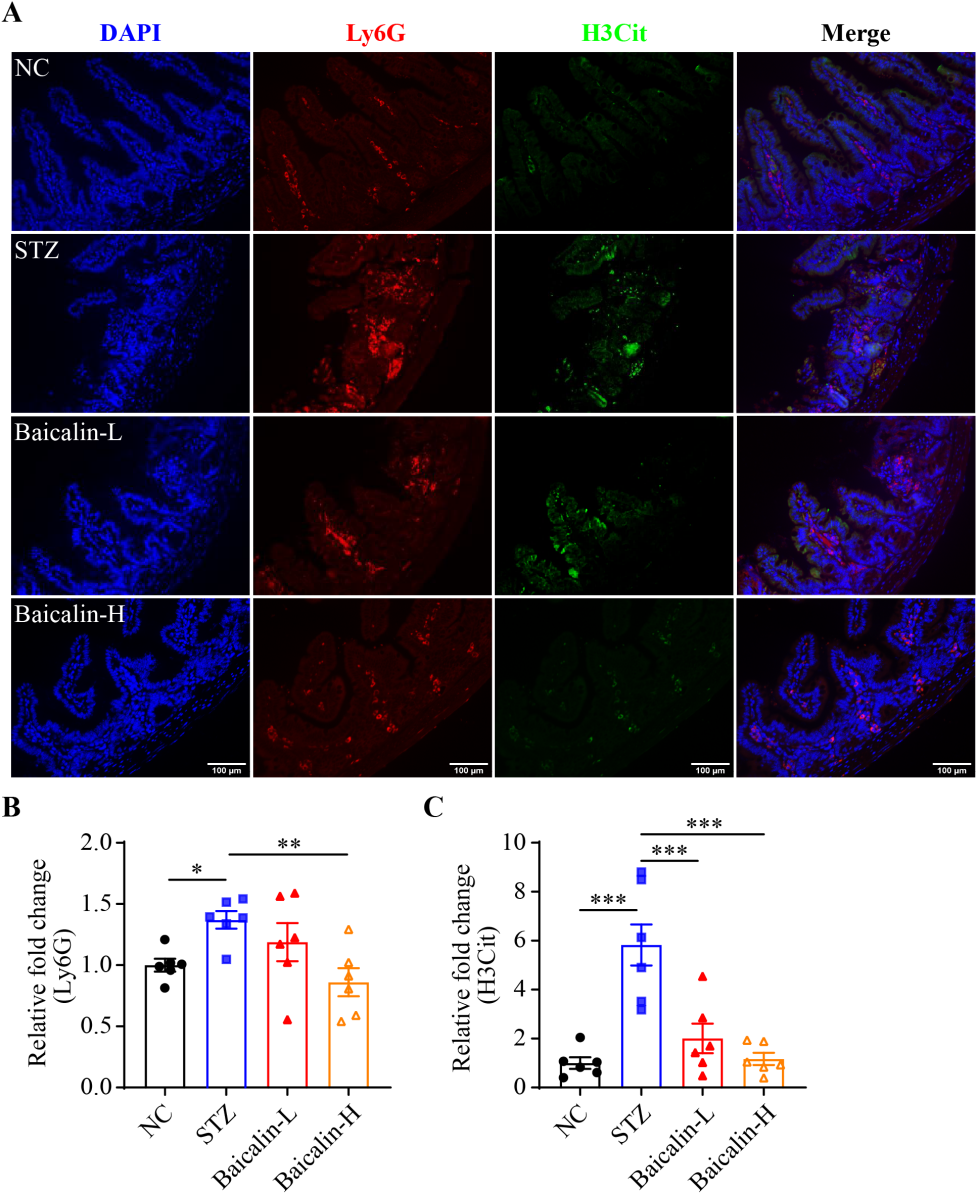
Baicalin attenuates hyperglycemia-linked intestinal NETs formation *in vivo*. **(A)** Representative micrographs showing Ly6G (in red) and H3Cit (in green) immunopositivity. DAPI was counterstained to visualize the nuclei (in blue). Scale bar, 100 μm. **(B)** Relative fold change in the Ly6G positivity (n = 6 per group). **(C)** Relative fold change in the H3Cit positivity (n = 6 per group). Data were expressed as mean ± SEM. * p < 0.05, ** p < 0.01, *** p < 0.001. NC, the vehicle-treated normoglycemic controls; STZ, the vehicle-treated STZ-induced hyperglycemic mice; Baicalin-L, the STZ-induced hyperglycemic mice treated with a daily dose of 240 mg/kg baicalin; Baicalin-H, the STZ-induced hyperglycemic mice treated with a daily dose of 1200 mg/kg baicalin.

**Figure 9:**
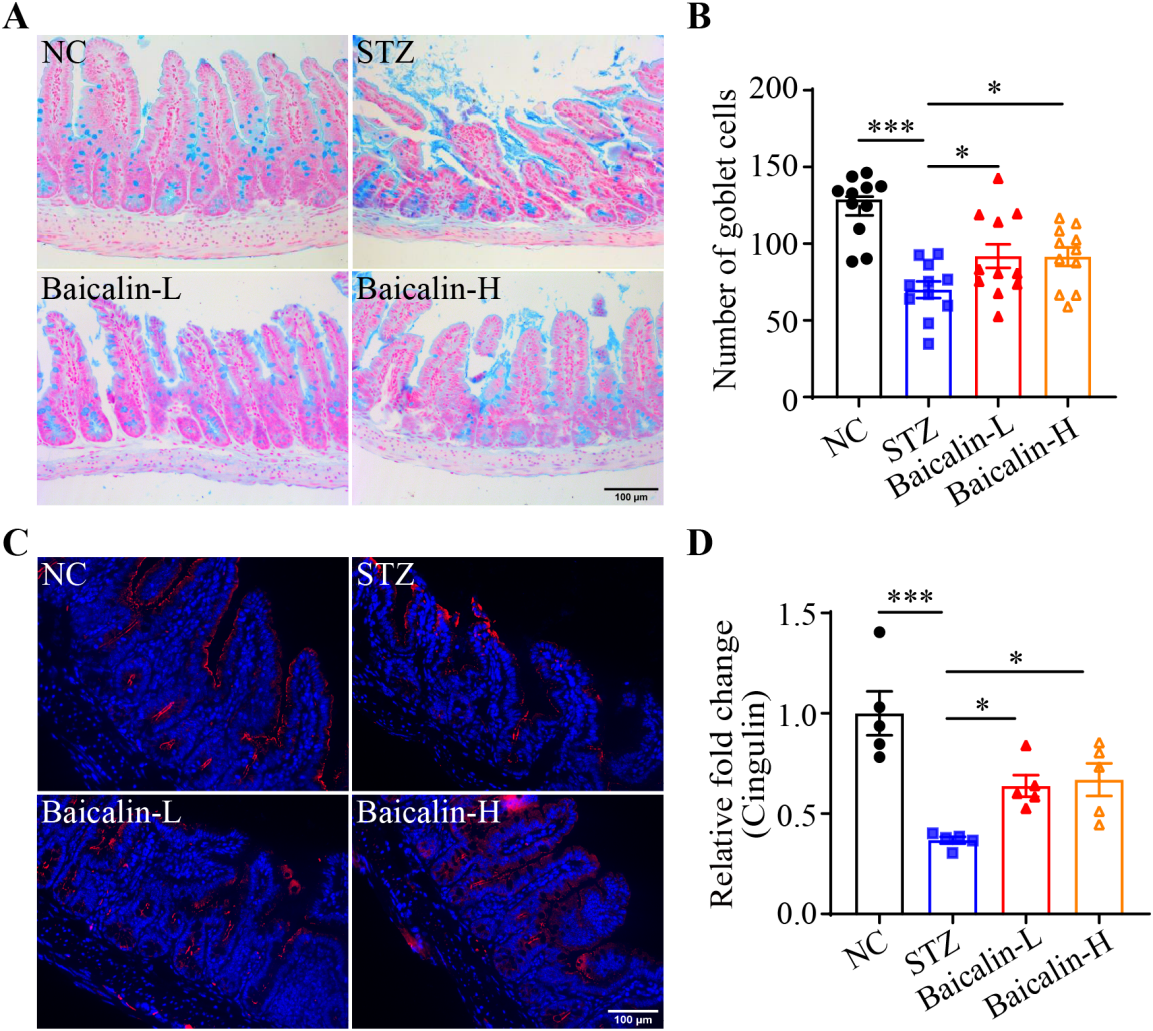
Baicalin protects against the intestinal epithelial barrier impairment in the hyperglycemic mice. **(A)** Representative micrographs showing Alcian blue-stained ileal goblet cells. Scale bar, 100 μm. **(B)** The number of the goblet cells per unit area (n = 11 per group). **(C)** Representative micrographs revealing cingulin immunopositivity in the ileal sections. Scale bar, 100 μm. **(D)** Relative fold change in the cingulin immunopositivity in the ilium (n = 5 per group). Data were expressed as mean ± SEM. * p < 0.05, *** p < 0.001. NC, the vehicle-treated normal controls; STZ, the vehicle-treated STZ-induced hyperglycemic mice; Baicalin-L, the STZ-induced hyperglycemic mice treated with a daily dose of 240 mg/kg baicalin; Baicalin-H, the STZ-induced hyperglycemic mice treated with a daily dose of 1200 mg/kg baicalin.

Next, the putative effects of baicalin at alleviating hyperglycemia-associated NETs formation was validated *in vivo*. Although no significant changes in the level of the fasting blood glucose were noted (**Supplementary Figure 4A**), significantly decreased Ly6G immunopositivity was observed in the STZ mice treated with high-dose baicalin compared to the mice receiving vehicle treatment (**Figures 8A, B**). Furthermore, both low-dose and high-dose baicalin treatments resulted in remarkably lower H3Cit immunopositivity in the ileum compared to the vehicle-treated STZ mice (**Figures 8A, C**). Histological examination further revealed improvement in the ileal morphology in the STZ mice that received both low-dose and high-dose baicalin treatment compared to the vehicle-treated STZ mice (**Supplementary Figure 4B**). Consistently, more goblet cells (**Figures 9A, B**) and increased cingulin immunopositivity (**Figures 9C, D**) were detected in the ileum from the baicalin-treated STZ mice compared to the vehicle-treated STZ mice. Taken together, these results support that baicalin is effective at attenuating NETs formation and intestinal epithelial barrier impairment in the hyperglycemic mice.

## 4 Discussion

**Figure 10:**
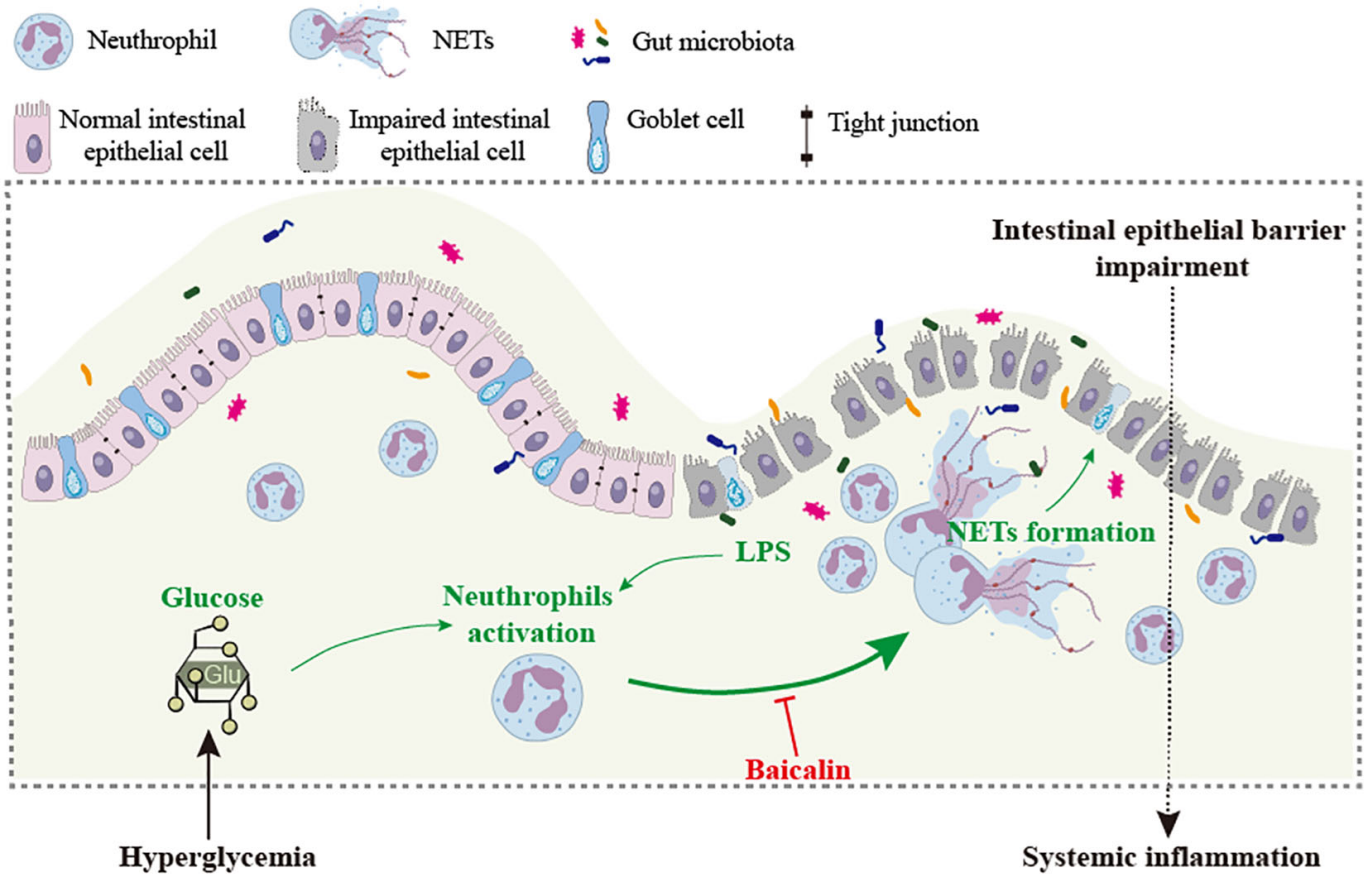
Baicalin mitigates hyperglycemia-linked intestinal epithelial barrier impairment in part via suppressing NETs formation. Intestinal barrier impairment contributes to hyperglycemia-associated systemic inflammation. NETs induced by high level of glucose and/or bacterial products such as LPS are directly involved in the pathogenesis of intestinal epithelial barrier impairment. Baicalin directly suppresses NETs formation triggered by high level of glucose, LPS or both, which may in part account for baicalin-conferred protection against intestinal epithelial barrier impairment under hyperglycemic conditions.

Although the associations between hyperglycemia and intestinal epithelial barrier impairment, hyperglycemia and NETs formation as well as NETs and intestinal epithelial injuries have been separately established, the causality of hyperglycemia-induced NETs formation in intestinal epithelial barrier impairment remains to be addressed *in vivo*. Therefore, one of the major aims of the current work was to clarify the mechanistic contribution of NETs to intestinal epithelial barrier impairment under hyperglycemic conditions. The *in vivo* findings here unveil that NETs are present in the intestinal epithelium in the hyperglycemic mice. DNase I treatment or *Padi4* gene deficiency abolishes NETs formation in the intestinal epithelium and mitigates intestinal epithelial barrier impairment in the hyperglycemic mice, supporting the causal relationship between NETs and hyperglycemia-associated intestinal epithelial barrier impairment. Based on these findings, we further demonstrate that baicalin suppresses NETs formation in the neutrophils stimulated by high glucose, LPS, or both. Most importantly, baicalin attenuates NETs formation and preserves the intestinal epithelial barrier integrity in the hyperglycemic mice (**Figure 10**).

First of all, our major findings here provide *in vivo* evidence supporting the mechanistic implications of NETs formation in the pathogenesis of hyperglycemia-linked intestinal epithelial barrier impairment. Impaired intestinal epithelial barrier plays an important role in driving the development of systemic inflammation under hyperglycemic conditions. However, the mechanisms accountable for hyperglycemia-linked intestinal epithelial barrier impairment remain to be fully elucidated. It has been demonstrated that high glucose directly stimulates NETs formation. Increased NETs formation promotes diabetes-associated impairment of wound healing and diabetic retinopathy (6, 9, 10). Consistently, our results here demonstrate that high glucose alone or in combination with LPS leads to increased NETs formation in the neutrophils. Our *in vitro* findings also confirm that NETs directly impair the viability of the intestinal epithelial cells in a dose-dependent manner. Of note, at the doses that are not detrimental to the survival of the intestinal epithelial cells, NETs sabotage the barrier integrity of the intestinal epithelial cells. Most importantly, *in vivo* evidence here uncovers that along with impaired epithelial integrity, NETs could be readily detected in the intestinal epithelium in the mice 2 weeks into hyperglycemia, suggesting that NETs formation and impairment of the intestinal epithelial barrier are early pathological events during the course of hyperglycemia. Eliminating the DNA components in NETs by DNase I treatment or abolishing NETs formation by genetic ablation of *Padi4* improves the intestinal epithelial morphology in the hyperglycemic mice. These findings collectively support the causal relationship between hyperglycemia-induced NETs formation and intestinal epithelial barrier impairment. Meanwhile, the *in vitro* and *in vivo* findings suggest the possibility that DNA is involved in driving the compromised survival and tight junctional impairment of the intestinal epithelial cells under hyperglycemic conditions. Impaired integrity of the intestinal epithelial barrier is of paramount significance to the development of diabetes-associated systemic inflammation (2). The findings here further support that NETs contribute to hyperglycemia-linked intestinal barrier disruption, supporting the possibility that aberrant activation of neutrophils could be therapeutically targeted to control hyperglycemia-associated inflammation.

Secondly, our work here underpins the pharmacological value of baicalin in attenuating hyperglycemia-initiated NETs-mediated intestinal epithelial barrier impairment. Baicalin directly suppresses histone citrullination and NETs formation in the neutrophils stimulated by high glucose, LPS or both high glucose and LPS. These *in vitro* findings support the possibility that the beneficial impact of baicalin on preserving the integrity of the intestinal epithelium under hyperglycemic conditions could in part be ascribed to its direct action at inhibiting NETs formation promoted by high glucose and/or bacterial products such as LPS. Although the mechanisms underlying high glucose-triggered formation of NETs remain to be fully elucidated, it has been shown that NADPH oxidase-mediated overproduction of reactive oxygen species (ROS) is involved in high glucose-induced NETs formation (6). Under platelet-free conditions, which is more relevant to our *in vitro* experimental setting, LPS induce NETosis via mechanisms implicating autophagy and ROS production (20). Therefore, overproduction of ROS appears to be a common mechanism underlying NETs formation stimulated by high glucose, LPS or both high glucose and LPS. It has been demonstrated that without affecting the assembly of NADPH oxidase, an essential step required for the activation of NADPH oxidase, baicalin inhibits fMLP or PMA-induced ROS production in human neutrophils, supporting its pharmacological activity in scavenging reactive oxygen intermediates (18). Moreover, baicalin lowers the activity of myeloperoxidase in the presence of fMLP (18). Myeloperoxidase is required for NETs formation as neutrophils from the donors with myeloperoxidase deficiency fail to form NETs in the presence of PMA (21). Both hyperglycemia and LPS exposure are associated with heightened level of myeloperoxidase activity (22, 23). Therefore, it is possible that the antioxidant property of baicalin may contribute to its effects at attenuating NETs formation in the presence of high glucose and/or LPS. Meanwhile, myeloperoxidase is likely a molecular target of baicalin in this process. Future studies are necessary to delineate the molecular mechanisms underlying the effects of baicalin at inhibiting NETs formation under hyperglycemic conditions. In terms of the potential mechanistic implications of myeloperoxidase, it is worth delineating if baicalin directly interacts with myeloperoxidase and/or how baicalin suppresses the activation of myeloperoxidase in neutrophils.

Additionally, there are several limitations associated with the current study. First, the histopathological features associated with NETs formation were not evaluated in the other segments of the small intestine in the hyperglycemic mice, thus it remains unknown if hyperglycemia has a broad impact on the morphological integrity of the small intestine and if NETs formation is involved. Second, the intestinal permeability assays, for instance, FITC-dextran leakage assay, remained to be conducted in the hyperglycemic mice to directly assess the pharmacological impact of baicalin treatment on the intestinal barrier integrity *in vivo*. Moreover, the impact of baicalin on intestinal barrier impairment-linked metabolic inflammation under hyperglycemic conditions remained unknown. Future studies are warranted to address these remaining questions.

Taken together, the work here demonstrates for the first time that NETs formation promotes intestinal epithelial barrier impairment under hyperglycemic conditions. Most importantly, our findings here shed new light on the pharmacological mechanisms of baicalin in protecting against hyperglycemia-linked intestinal epithelial barrier impairment, which in part implicates direct effects of baicalin at suppressing NETs formation. The novel understanding of the mechanisms of actions of baicalin justifies its potential as an adjunct agent in controlling hyperglycemia-associated inflammation.

## Data Availability

The original contributions presented in the study are included in the article/**Supplementary Material**. Further inquiries can be directed to the corresponding authors.
