# Supplementary material for: Baicalin mitigates hyperglycemia-linked intestinal epithelial barrier impairment in part by inhibiting the formation of neutrophil extracellular traps
Source: Front Immunol. 2025 Mar 3;16:1551256. doi: 10.3389/fimmu.2025.1551256 (PMC11911346; doi:10.3389/fimmu.2025.1551256)
Supplement: Supplementary file 1 [file DataSheet1.docx]

Supplementary Material

**Supplementary Figure 1**


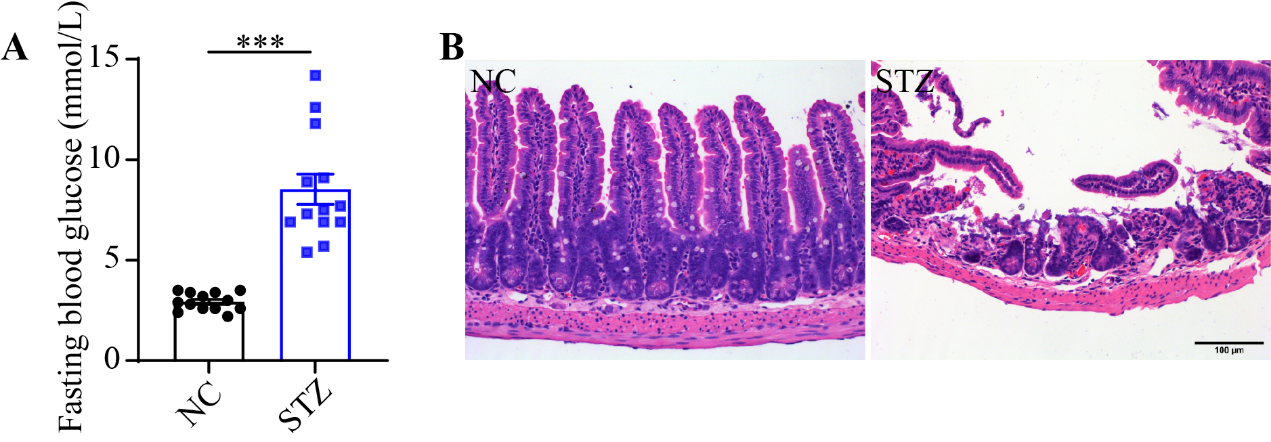


**Supplementary Figure 1. Hyperglycemia is linked to intestinal epithelial barrier impairment. A.** Fasting blood glucose (n = 13 per group). **B.** Representative micrographs showing ileal morphology as revealed by HE staining. Scale bar, 100 μm. Data were expressed as mean ± SEM. *** p < 0.001. NC, normoglycemic controls; STZ, STZ-induced hyperglycemic mice.

**Supplementary Figure 2**


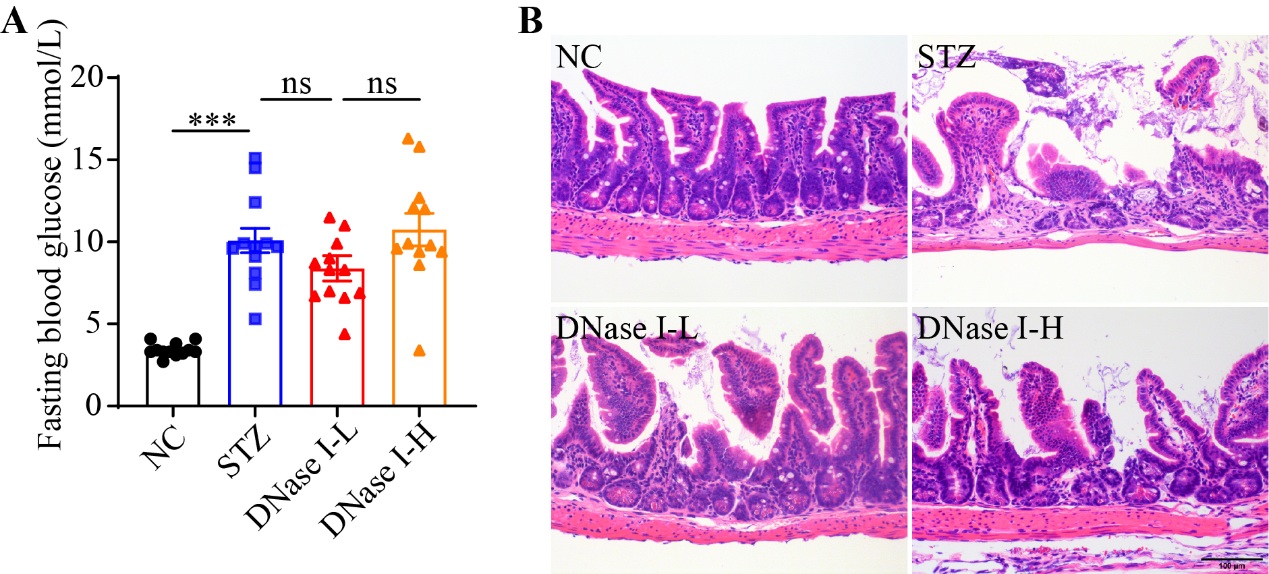


**Supplementary Figure 2. DNase I treatment ameliorates the intestinal epithelial morphology in the hyperglycemic mice. A.** Fasting blood glucose (n = 12 per group). **B.** Representative micrographs showing ileal morphology as revealed by HE staining. Scale bar, 100 μm. Data were expressed as mean ± SEM. *** p < 0.001, ns, not significant. NC, the vehicle-treated normoglycemic controls; STZ, the STZ-induced hyperglycemic mice treated with the vehicle; DNase Ⅰ-L, the STZ-induced hyperglycemic mice treated with a daily dose of 2.5 mg/kg DNase Ⅰ; DNase Ⅰ-H, the STZ-induced hyperglycemic mice treated with a daily dose of 10 mg/kg DNase I.

**Supplementary Figure 3**


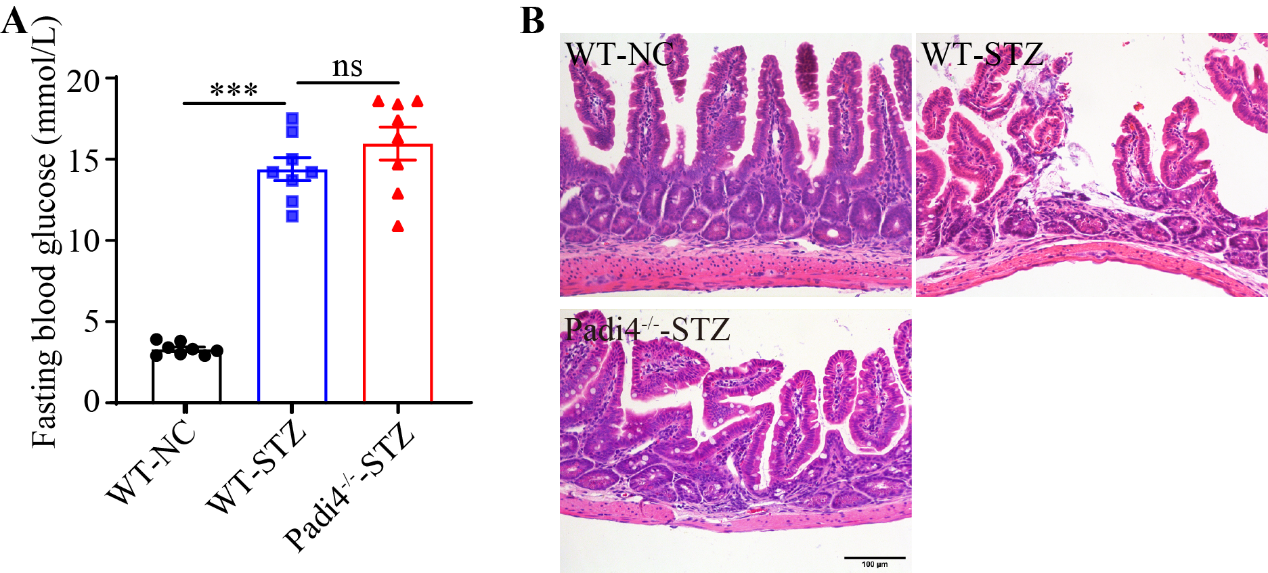


**Supplementary Figure 3. *Padi4* gene deficiency improves the intestinal epithelial morphology in the hyperglycemic mice.** **A.** Fasting blood glucose (n = 8 per group). **B.** Representative images showing the ileal morphology as revealed by HE staining. Scale bar, 100 μm. Data were expressed as mean ± SEM. *** p < 0.001, ns, not significant. WT-NC, the wild type normoglycemic controls; WT-STZ, the wild type mice subjected to STZ-induced hyperglycemia; *Padi4*^-/-^-STZ, the *Padi4*^-/-^ mice subjected to STZ-induced hyperglycemia.

**Supplementary Figure 4**


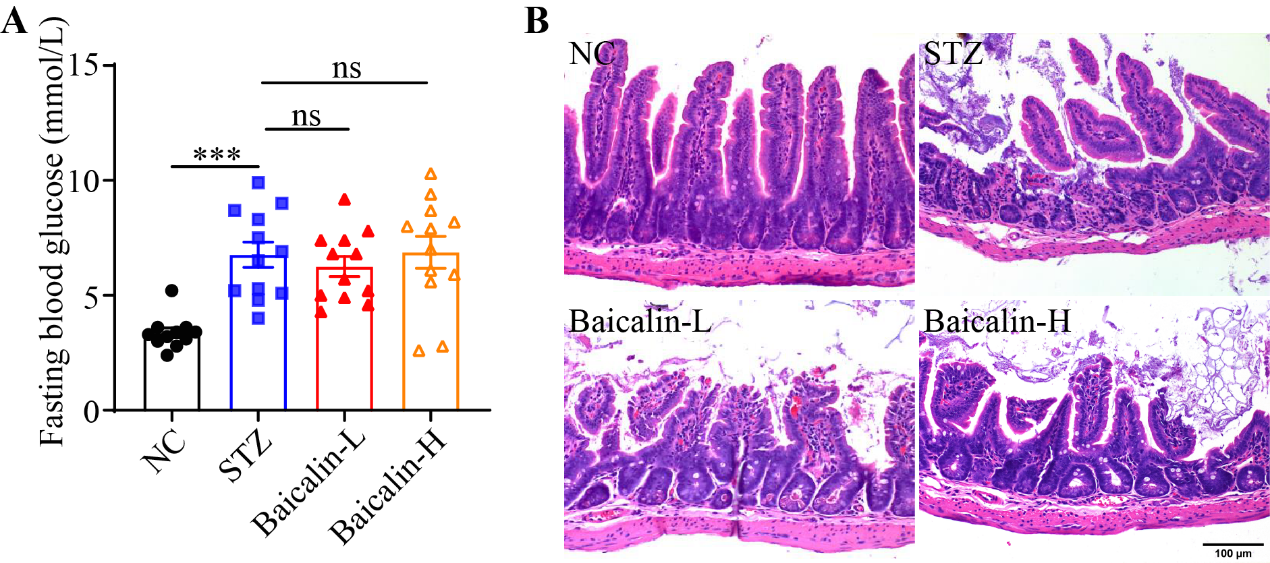


**Supplementary Figure 4. Baicalin partially preserves the intestinal epithelial morphology in the hyperglycemic mice. A.** Fasting blood glucose (n = 12 per group). **B.** Representative images showing the ileal morphology as revealed by HE staining. Scale bar, 100 μm. Data were expressed as mean ± SEM. *** p < 0.001, ns, not significant. NC, the vehicle-treated normoglycemic controls; STZ, the vehicle-treated STZ-induced hyperglycemic mice; Baicalin-L, the STZ-induced hyperglycemic mice treated with a daily dose of 240 mg/kg baicalin; Baicalin-H, the STZ-induced hyperglycemic mice treated with a daily dose of 1200 mg/kg baicalin.
